# The Hospital. Nursing Mirror

**Published:** 1898-04-09

**Authors:** 


"The Hospital" Nursing Mirror.

Being the Nursing Section of "The Hospital."

[Contributions for this Section of "The Hospital" should be addressed to the Editor, The Hospital, 28 & 29, Southampton Street, Strand, London, W.C., and should have the word "Nursing" plainly written in left-hand top corner of the envelope.]

### News from the Nursing World.

### THE QUEEN AND HER SOLDIERS.

Once more our Gracious Sovereign has expressed her sympathy towards her wounded soldiers in a manner all can understand and admire. To each of the 19 wards at Netley Hospital visited by the Queen just before leaving England, Her Majesty has sent a photograph of herself, handsomely framed in oak and gold, and bearing the following inscription: "Presented by Her Majesty the Queen, 1898." She has also sent a fine portrait of the Prince Consort, and engravings of herself in her pony carriage; of the Jubilee procession passing Trafalgar Square, the Jubilee procession leaving Buckingham Palace; a group of the Royal Family, one entitled "The Queen and the Empire," and three coloured prints called "Farewell," "The Story of an Elopement," and the "Thin Red Line." All these pictures are framed in the same manner as the photograph, and bear the same inscription. The Queen has also written to Major-General W. Nash, M.D., who at her request has obtained the very finest artificial limbs procurable for the mutilated soldiers. Her Majesty has given to the hospital four invalid couches of the latest pattern, which will prove most comfortable for the use of the convalescents. The Princess Henry of Battenberg has sent a fine steel engraving of the late Prince.

### THE PRINCESS CHRISTIAN AT NICE.

H.R.H. Princess Christian, on behalf of the Queen, and accompanied by Princess Henry of Battenberg, last week inaugurated the new Victoria ward of the Foreign Hospital at Nice. The nursing staff and the ladies of the Hospital Committee were presented to their Royal Highnesses, and the religious portion of the ceremonial was performed by the Bishop of Ripon. The Princesses visited the wards at the conclusion of the ceremony.

### A STRIKE AT A HOSPITAL.

The committee of the Llanelly Hospital are confronted with the difficulty of re-staffing their institution. For some time past there has been considerable irritation because, it is alleged, the matron has not been given entire management of the nursing and domestic staffs. Full particulars will doubtless be forthcoming in due season of the deadlock, which has resulted in the resignation not only of the matron, but of the nurses and servants. The committee has accepted the resignations, and has called a special meeting to consider the position and to take steps for supplying their places.

### THE EVOLUTION OF A STATE HOSPITAL.

An informal ceremony marked the opening of the new nurses' home at Keighley Infirmary. This home, just built and furnished at a cost of £1,000, is detached, and affords accommodation for eight nurses, providing each with a separate bedroom. It also contains a dining-room, a room for the superintendent nurse, a kitchen, bath-room, and servants' room. Alderman Clough gave a short history of the progress of the infirmary. At first it was under the charge of a porter, his wife, who acted as cook, and the paupers, who, with her aid, nursed each other. There is now a superintendent nurse, at the head of a staff of six trained nurses, the sanitary arrangements have materially improved, and the results have been highly satisfactory from a medical point of view. Miss Cockshott expressed a wish that the infirmary would, in time, be regarded as a State hospital, and not as a workhouse institution. The services of Miss Heath, the superintendent, and her nurses were referred to in eulogistic terms, and a vote of thanks to Mr. Conchar, chairman of the committee, closed the proceedings.

### A BIT OF BUTTER.

The value of a bit of butter rarely strikes those who are lucky enough to use just as much as they want. To many nurses in institutions, however, the case is different, and they doubtless indulge in an occasional grumble at the meanness in trifles of those in authority over them when the allowance is short of their desires. What is a trifle from the nurse's point of view is a serious consideration, however, to the authorities. Here is a recent example: Not very long ago a well-known matron of one of our largest London hospitals expressed her wish to add half an ounce of butter each to her nurses' breakfasts. Her chairman agreed with her that it was a desirable innovation, and the cost was immediately counted. It reached the large sum of £160. So the nurses must do without their extra butter until subscriptions come in that would justify the expenditure.

### THE DAUGHTERS OF THE KING.

The Daughters of the King is the title by which a new order in the Anglican Church is called. For some time this guild has been flourishing in America, and it has been transplanted to England under the auspices of the clergy and bishops. The aim of the society is the banding together of women for the conversion and help of other women, and there are rules of prayer and service as well as a religious ceremony of initiation. The members wear a badge, and promise to bring at least one other woman within the sound of the Gospel message every week. Thus, it will be seen that it is more missionary in its scope than its forerunners. For ourselves, we cannot see the need of adding ceremonies and regulations to the simple faith to which we were admitted at our baptism, but there are those who find such guilds helpful, and undoubtedly such organisations are useful when movements requiring widespread co-operation amongst women are on foot. We trust, however, that any nurses that may join it will remember that their duty in life is to care for the sick, and not to become proselytising agents. They will do well to note that the aim to spread Christ's kingdom on earth, and to induce other women to attend Divine service, is a very different thing from perpetually forcing certain religious tenets on their patients.

### AN UNHOLY ALLIANCE.

A week or two ago we touched upon the topic of nurses selling by commission to their patients. A far graver charge has been brought against nurses by a Berlin physician, one which he declares is half a century old. He alleges that the nurses in private hospitals in that city and in others are in league with the undertakers, who give as much as £5 commission for a good job, and suggests as a remedy the employment of women working in sisterhoods, or who are actuated by higher motives than that of gain. Undoubtedly there are black sheep in every flock, and it is no use shutting one's eyes to the fact that all are liable to err if tempted. Nothing can be done for black sheep but to expel them from the ranks of whatever society they may belong to, but much may be done for the rest by minimising the risks of temptation. Adequate remuneration, due provision for sickness and age, strict prohibition of questionable gains and perquisites by all nursing co-operations and societies, are powerful safeguards against such unholy alliances as the one we notice. We believe, also, that the majority of British nurses have as righteous a horror of such practices as anyone, and will resolutely discountenance all attempts on the part of tradesmen to introduce in England so baleful a custom.

### BRISTOL DISTRICT NURSES' SOCIETY.

It is now no longer considered only desirable that a neighbourhood should have nurses for the sick poor, but the district that has not one is commiserated upon the fact and money is collected to supply the lack. The needs of the Two Mile Hill, Bristol, were brought prominently forward at the annual meeting of the Bristol District Nurses' Society. With a population of 9,000, only 1,000 had the benefit of a nurse. Sickness has been rife in this neighbourhood, for work has been scarce and semi-starvation prevalent. The society does its work economically, the average cost of a visit being sixpence. During the year the nurses have paid 70,000 visits, and the expenditure has been £1,600. The society is helped by the Laura Edwards Memorial Fund, which is most useful in enabling both women and children to be sent away for change. The needlework guild, too, greatly strengthens the nurses' hands by providing warm clothing and comforts for the sick. We are sorry to note that the earnings of the private nurses are not kept separate from those of the district nurses, Miss Errington, the hon. secretary, stating that "The private nurses' earnings have much increased and have proved a great help to the funds." We trust that the committee will see their way to make the benevolent part of their institution self-supporting, and to give to the private nurses the full equivalent of their earnings, less, of course, expenses.

### MALE NURSES (TEMPERANCE) CO-OPERATION.

The work of the male nurse is becoming more decidedly recognised and appreciated. Some four years ago the Male Nurses (Temperance) Co-operation was begun at 10, Thayer Street, Manchester Square, W., and during the past year the average earnings per nurse have been £102 3s., whilst the secretary has frequently received applications from provincial hospitals for men with asylum experience for employment generally and in the male wards. The staff has also been increased, and a sick fund established, though luckily the latter has only had to be drawn upon to the extent of 8½d. per member. This growing inquiry for trained male nurses emphasises the need of training schools for men. Sooner or later provision must be made to meet the demand, and it is uncommonly shortsighted not to march with the times instead of lagging behind. There can be no possible danger of male nurses ousting women from the profession, for nursing is essentially a womanly art, but there are cases which require masculine strength, and there are duties which may be more fittingly performed by a man than a woman, even though a woman discharges them unflinchingly if necessary, and it is fair to ask why should men not be taught to do them if they wish it?

### THE MISSIONARIES AND THE PLAGUE.

An article in the *Zenana* for April gives an interesting account of the volunteer work of missionary ladies in nursing the plague. It is impossible, says the writer, the Rev. A. R. Cavalier, to realise the extent and severity of the sufferings which this visitation is causing without going amongst the people and seeing it. The streets of busy towns are silent, the doors are locked, and the houses unroofed in order to let in the sunlight and air. At Malegaon, within a few days, 20,000 poor folk were turned out of doors by the orders of the Government, and were camped out in small straw huts, some having lost everything, as the furniture and effects of infected houses were burned. Amidst this grievous distress, as on former and similar occasions, the Europeans have been most kind, doing everything in their power to alleviate the hardship of the populace and protect them against the oppression of their own countrymen. Two lady missionaries, Miss Clark and Miss Fulcher, in this place, have during the last four months given themselves up to nursing the sick. At Nasik, with a population of 25,000, the same distressing state of things prevailed, and Miss Harvey, a missionary, volunteered and is doing splendid work. At Poona, now happily nearly free, there were no less than nine red crosses on one hut, showing that nine victims had become infected and died of the plague in it.

### SHORT ITEMS.

The festival ball in aid of the Italian Hospital is to be held on May 2nd, under Royal patronage, at the Galleries of the Institute of Painters in Water Colours, Piccadilly. Miss Bianconi, the hon. secretary, 22, Russell Square, will be glad to answer all inquiries.—The Worthing Nurses' Association have to lament the loss of their first president, the Dowager Lady Hatherton. Lady Fletcher has consented to take her place. The committee has decided to put the Diamond Jubilee contributions in the Post Office Savings Bank.—It was resolved at the annual meeting of the Crompton Nursing Association that the services of a second nurse for infectious cases ought to be secured, as well as an ambulance carriage. The Rev. D. Morgan was at the same meeting elected president in room of the Rev. Josiah Evans, whose health, unfortunately, compels his withdrawal from the position.—Miss Gethen is lecturing weekly to a large class at Bexley, Kent. — Miss McDougall, whose sad death from plague at Bombay is in everyone's memory, was a St. Bartholomew's nurse, where she obtained the gold medal in her final examination.—The committee of the Barrhead District Nursing Association (Glasgow) find the work too much for Nurse Milne without assistance. As the income is £50 in excess of expenditure, slightly more liberal contributions will make up the required sum to provide another nurse.

### Lectures to Surgical Nurses.

By H. A. LATIMER, M.D. (Dunelm), M.R.C.S. of Eng., L.S.A. of London, Consulting Surgeon, Swansea Hospital; President of the Swansea Medical Society; Lecturer and Examiner of the St. John Ambulance Association, &c.

### XXVIII.—SIMPLE ANATOMY OF HERNIA—HOW TO RECOGNISE GRAVE CONDITIONS OF RUPTURE—PREPARATIONS FOR THE OPERATIONS OF HERNIOTOMY AND LAPAROTOMY; DIETARY AND SUBSEQUENT TREATMENT.

I NOW turn to consider a surgical complaint which is constantly being met with in one form or another, viz, hernia or rupture. Strictly speaking, a hernia is "the protrusion through an opening of part of an organ from its natural cavity," so that if a portion of the brain squeezes its way through the membranes which cover it, or some of the lung protrudes through the pleura which encloses it, there is hernia: but the word, unless qualified by a statement of what organ is concerned in an escape—as hernia of the brain, hernia of the lung—is only used when some portion of the bowels has protruded through its natural coverings. This they are apt to do very frequently when pressure is exercised upon them during violent muscular action in which the walls of the abdomen are made rigid—as during the act of coughing, straining to pass water, or in the closet, in blowing wind instruments, or lifting heavy weights—for then all the abdominal walls are kept rigidly contracted, and the intestines are pressed upon in all directions, with the result that if there is a weak point anywhere some portion of bowel which lies against it forces its way outwards, and, pushing its natural coverings before it, escapes from the cavity which is its home. There are various weak points in the abdominal wall left there after the escape of organs from the abdomen, or existing at places which are pierced by ligaments or bloodvessels. These openings have natural coverings, but the coverings are prone to yield to pressure from within. It is quite unnecessary to tell you all the places where hernia may occur; the chief regions are the groins and the navel, because here especial weak points exist. When a hernia has formed here it betrays itself as a swelling under the skin, which, if the escaped bowel has not become gripped or stuck to the parts around it, disappears when the patient lies down or presses upon it, for now the bowel has slipped back into the belly. A hernia which behaves in this way is described as being "reducible," and the bowel, or omentum, which constitutes it, should be kept from again escaping by its owner wearing an instrument called a truss, which consists of a band encircling the lower part of the body above the hips, and having an appropriately-shaped pad, which presses upon the weak spot where the hernia has shown itself, or by an operation for radical cure being performed. If well fitting trusses are worn continually, by day and by night, in such cases, the person suffering from a hernia is safe; but if, with such a defect, a person neglects to avail himself of this protection, the bowel, or the layer of fat which lies near it, which is called the omentum, pushes its way through the opening in larger and larger amounts, and is liable at any time to be fixed in its new position, to undergo inflammation, and to be blocked, and so to prevent the passage of its contents through its channel; and as this inflammation speedily spreads to its covering of peritoneum—called its sac—which it has pushed before it in its escape from the abdomen, there quickly ensues that painful and dangerous disease called peritonitis. A hernia lying in this unreducible condition is described as being "strangulated," and unless pressure is quickly taken off it by its return to the abdomen, or by an operation, it quickly inflames, becomes gangrenous, and kills its victim. You should be able to recognise a strangulated hernia so as to know what grave importance is attached to its early recognition, for unless the surgeon can manage to operate upon it within forty-eight hours after complete obstruction of the bowel has set in, it is very likely to prove fatal to the patient. I have operated at longer intervals than the above and with success, but there is no doubt that the sooner the strangulation is relieved the greater chance there is of saving life. These are the symptoms which mark a strangulation: Great pain in the abdomen, of a colicky kind, felt all over it, but especially in the neighbourhood of the navel; complete obstruction of the bowels, not even wind escaping; vomiting, which is severe and frequent from the start; anxious expression of face; dry, brown tongue, and small pulse. The matters brought up during vomiting change in character. At first they only consist of whatever food may happen to be in the stomach; then, after this, bile and bile-stained mucous are ejected, and as the strangulation remains unrelieved, a horribly offensive brown vomit, smelling like a bad motion, is brought away. Be very careful at all times to save these vomited matters for the surgeon's inspection. Whatever doubts he may have had as to the nature of the case, during the periods of early vomiting, are resolved at once when he sees this fæcal vomit—known as "stercoraceous vomit"—for now he has a sign presented to him pointing out definitely that somewhere or other there is obstruction of the bowels. He will have been sure of this already if, in one of the usual situations for a hernia, he has found a swelling which cannot be returned to the abdomen, and which does not give any impulse when the patient coughs; but it may be that the obstruction is situated somewhere in the interior of the belly, out of sight and touch, and yet the symptoms will be just the same, and will require the use of the knife in all probability before they can be relieved. When the hernia can be felt it is customary to try to return it by squeezing it in a skilful manner, and sometimes this application of the taxis, as it is called (and especially if done when the patient's muscles have been relaxed by his being put under the influence of an anæsthetic), is successful in righting matters; but in the greater number of cases an operation has to be performed, in which the bowel is cut down upon, and the material—whatever it may be—which is gripping or strangulating it, is divided. This operation, when applied for the relief of an external rupture, is called "herniotomy;" when for the relief of an internal obstruction is called "laparotomy" or "abdominal section." You will have to prepare the patient for these by carefully washing the abdominal walls, and by cleansing them with an antiseptic lotion. During the whole of the illness the diet will have consisted of very small quantities of iced soda-water and milk, or of a beef juice, or of ice alone. After the operation has been performed, the patient will be placed on his back in bed, with his knees drawn up and supported by pillows; whilst, to take off the pressure of bedclothes above him, a large cradle will be placed over his abdomen. For twenty-four hours it is well to give as little of anything by the mouth as is possible—at the most, one teaspoonful of fluid every half-hour. If there is intense thirst, much relief will be afforded by allowing the patient to rinse his mouth out with hot water. After this time small quantities of weak arrowroot gruel, tea and milk, or milk and soda-water may be given; and from the third day, if all is going well, animal broths, or even a little thin bread and butter, may be taken. On no account, in any of these cases from the start to the finish, are you to give an aperien This rule is absolute, and must not be departed from without the advice of the surgeon in attendance. Many of these cases would never assume the importance they do if this plain rule were borne in mind. The right method of relieving the bowels in all cases of obstruction is by the use of enemata. They should be relieved from below; for if aperient medicines are given by the mouth they only increase the mischief, already present, by causing attempts at active movement in the muscular walls of the bowels, and by driving the plug of fæcal matter more firmly into the seat of obstruction than ever. Being engaged in telling you of these abdominal operations I shall do wisely, I think, if I finish with this the consideration of this branch of surgery before passing from it; so I will conclude this lecture with some references to another class of diseases affecting organs within the abdomen. Of late years, and especially since the advent and development of the antiseptic system in surgery, a great number of diseased conditions of abdominal organs which were formerly regarded as fatal ones, have been treated by surgeons with the greatest and most encouraging effects; and it is no exaggeration to say that the general expectation of life has been vastly extended by the successes which have attended this branch of surgery alone. Not only have diseased portions of the hollow viscera been removed, and the remaining healthy portions been stitched together to allow of a restoration of their continuity being established, but the more solid organs, such as the spleen and kidneys, have been cut into for the removal of stones, or have themselves been taken away altogether. The stomach, the gall bladder, and the urinary bladder have been freely incised, and products of disease have been withdrawn from their interiors, and all these operations have been performed with a constantly increasing measure of success, as experience has ripened and methods have improved. Generally speaking, the nursing work you have to do in these kind of cases runs in the lines I have just been speaking of, and the dietary laid down for them is of the type recommended in the treatment after operations for the relief of intestinal obstruction.

### Nurses and Note-Books.

By a Trained Nurse.

Everybody makes good resolutions and everybody occasionally breaks them—and this to such an extent that the ultimate fate of these broken vows has become proverbial. The habit of diary keeping is one frequently formed in the rush of good resolutions, and as often as not is abandoned even in the earlier months of that year which has initiated the good habit.

The hospital nurse should keep a diary—a professional one—with scrupulous conscientiousness. Day by day she should enter any practical "wrinkle" obtained by her own invention, or observation, or through the inventiveness of others. In a year such a book would be of infinite value, in ten years the volume would be of priceless worth. The publication of such a book—duly edited and revised—would form a compendium of nursing hints such as it is impossible for a nurse-pupil to obtain for love or money. For the average text-book is written out of the wealth of theory rather than out of the fulness of practice. The nurse must therefore supplement her library by the making of books for herself, and no better education in nursing could be devised than the exercise of such a faculty.

The private nurse should keep two diaries—a professional and a domestic. And the keeping of these may be a very simple matter, entailing only a few daily minutes, so that the private nurse must not be appalled at a suggestion which at first sight might seem to involve a good deal of labour.

It is customary nowadays for people to smile at the word "domestic," for domesticity in woman is waning, and has been declared derogatory and out of fashion. It is not necessary here to discuss the merits of such a decree beyond saying, if it be believed by any so much the worse for women and the homes over which they preside. But despite the fact that modern femininity devotes most of its energies to acquiring knowledge and habits which have no home value, it is impossible for a private nurse to enter the many strange and fresh households with which she comes in contact during even a year of her life without picking up domestic knowledge which might prove of infinite value in her profession were she to remember it all. Each housekeeper, even if she be an inefficient one, knows a few fresh dishes suited to the sick, has some domestic "wrinkle" or other which should be jotted down in the domestic diary for future use. In two or three years a most valuable accumulation of useful knowledge would thus be obtained, that kind of everyday practical information which books cannot and do not impart. Each house has its own bit of domestic wisdom—its own unwritten lore of the culinary art—one new dish, at least, which would prove a worthy addition to such a domestic encyclopædia, a book which every private nurse could compile without difficulty or mental strain.

Much really valuable knowledge is lost and forgotten beyond redemption, all for the want of a sixpenny scribbling book and an indelible pencil. To mention one of the many ways such a domestic diary would prove of the utmost practical use to a private nurse, the list which she might make of convalescent dishes would in itself recommend the keeping of such a book. However conversant a nurse may be with the cookery of the sick-room proper—and by-the-bye very few nurses indeed are past masters of this invaluable art—there is a whole army of nurses who invariably suggest a boiled sole as the first solid food for a convalescent. And here the commissariat suggestions come to an abrupt ending. They begin with the sole and end with it. A peep into her private book would make her the mistress of the dietary situation. Again, there are nurses innumerable who have most vague notions of the seasons of various foods, and will suggest salmon or oysters at a time when the purchase of such viands would constitute a legal crime. Few—very few nurses—are familiar with this most important branch of housewifery, the knowledge of when special foods are at their very prime. But the domestic diary, if properly kept and tabulated, would remind them in a moment of what was "in" or "out of season," so that the lamentable mistakes made on this point might be avoided. Hospital training and the living in "Nurses' Homes" is so serious a drawback to the individual, so far as her housewifery capacity is concerned, that it behoves the nurse to keep careful watch and ward over herself, lest she lose interest in kitchen details and housekeeperly routine. It is a good plan, therefore, to keep an eye on the shops and notice the various foods in season, so that one may not suggest—as a nurse recently did—that "a little bit of grouse would be nice for the invalid"—and this in May!

A private nurse living in rooms and housekeeping for herself is apt to bound her cuisine by chops and steaks and eggs, so that unless she keeps such a diary as is suggested, she is apt to prove very much at sea when called upon to suggest some dainty dishes for a convalescent who has been very much accustomed to "the good things of this life." A few good recipes, some timely hints as to "seasons," the grouping together of light and suitable dishes to form attractive and seasonable menus for the sick, will prove of such timely assistance in hours of housekeeperly difficulty that no nurse having once started a domestic diary will be tempted to re linquish it, for its value will so soon recommend itself to her affections. Many private nurses keep an elaborate case-book, wherein all the medical details of their successive patients are entered, and the drugs and prescriptions which entered into the cure are faithfully recorded. But not a single word is mentioned as to the formula of that excellent beef-tea or those delicious peptonised oysters which helped him so enormously through his hours of need—an omission very curious to the student of humanity who expects, but by no means finds, that such domestic details are a kind of second nature to a woman.

By all means let every trained nurse keep a case-book and a volume devoted to the entry of all nursing details and fresh hints thereon. For every doctor she works with will have some admirable hints to give her as to nursing, and she will come across many desirable old woman's wrinkles and practical useful subtleties for the sick room among the friends and families of her patients. But she must never forget that the domestic side of nursing is one of its most valuable attributes, and she should therefore bear in mind that the knowledge held in her domestic diary will be as valuable—and perhaps even more valuable—to the sick people under her care as that contained in her professional "case-book."

### Antiseptics for Nurses.

By a Medical Woman.

### VII.—PREPARATION OF THE OPERATING ROOM—STERILISATION OF INSTRUMENTS.

If it is necessary to have an operation in a private house, the preparation of the room should engage the most anxious attention of the nurse. If sufficient time is given, and those concerned are willing, curtains should be taken down, and if of washing material, should be washed and put up again clean. If they are of woollen material it would be well, if possible, to replace them by washing curtains, which can be of a quite inexpensive kind. If the bedstead is wooden this should, if possible, be replaced by an iron one; but if this is impracticable, the nurse should see that the wood is thoroughly scrubbed with a disinfectant, such as 1 in 500 perchloride of mercury or 1 in 20 carbolic, and she should specially see that no dust lurks in any corner or ill-fitting join. If she can have curtains and valance dispensed with so much the better, but probably those who retain wooden beds will cling to curtains, &c., and the most the nurse can do is to secure these being perfectly clean for the occasion; mattress and bedding, also, must be the most suitable that can be procured. The carpet should be removed, the walls and ceiling dusted down, as well as all the things in the room, and these preparations should be at least carefully superintended by the nurse, even if she does not do them herself. All preparations should be completed the day before the one appointed for the operation, so as to allow any dust in the air to completely settle. Nothing could be more undesirable than to prepare the room the very morning of the day of operation, but the floor should be rubbed over last thing with a damp cloth wrung out of antiseptic lotion.

Having made the room as aseptic as possible, the nurse should have ready the means for sterilising the instruments which the surgeon will bring with him. This is easily done if a new tin pan, or, better still, a fish kettle, oblong in shape and not too large, can be procured, and a wire tray or basket to fit into it. This should be filled with a sufficient quantity of hot water, to which either salt or common household soda to the amount of 1 per cent. should be added; the pan should be on the fire, and the water quite boiling by the time the surgeon arrives, so that the instruments can be put into the basket at once and boiled for five minutes. This is the simplest and most effectual method, and as the saucepan or fish-kettle is not injured by the proceeding it can generally be procured without trouble in a private family, and in every cottage hospital such should be kept entirely for this purpose, and the nurse who has the care of the instruments need never fear infection from various wounds, even suppurating and highly infectious ones. Not long ago it was thought quite sufficient to put the instruments into carbolic for a short time before they were required for an operation, without previously sterilising them. The carbolic, however, tends to blunt the edges of knives if they remain in it for long, and the hands of those who have to handle the instruments suffer considerably. Some persons are very sensitive to the effect of carbolic, which causes eczema in some, and in others some constitutional effects, whilst in all the skin is macerated and liable to crack. Carbolic is still used for the instruments in the majority of hospitals, but it is to be hoped that sterilised water will soon be universally substituted for carbolic, since the instruments, being already thoroughly clean and sterilised, have only to be kept in a fluid free from germs and protected from particles of dust that might adhere to them. It is useless to try and cleanse with carbolic those instruments that are much soiled with pus, fat, or blood. These must be thoroughly scrubbed with a brush and plenty of soap and hot water, and can then, after boiling, be kept in carbolic or sterilised water.

That celebrated German surgeon, Bergmann, gives the following directions for cleansing instruments: "After the operation, first rinse the instruments in ordinary water, then lay them in a hot solution of soda and soap, and scrub carefully and energetically in every part with a brush. Then rinse them again, and clean them with alcohol and a piece of wash-leather. Again rinse them once more in a solution of soda, and carefully dry them. All coarser contaminations are thus got rid of, but even then some germs will adhere, the number depending on whether the instruments are difficult or easy to clean, are smooth, or have many corners or niches." The different means for sterilising instruments that have been proposed are hot air, steam, and boiling water or other fluid.

Hot air has been repeatedly and strongly advocated, but it has three great and insuperable disadvantages, viz., it takes too much time, it is a very clumsy method, and hot air rusts steel instruments. Attempts have been made to shorten the time by using an apparatus which can be very quickly heated, but it has been found that there is a considerable difference in the temperature at the bottom and at the top of the apparatus, and consequently this plan is not satisfactory. The rusting of the instruments is said to be prevented by properly ventilating the apparatus, since it is during the cooling-down that moisture is deposited and causes rust.

Steam certainly disinfects more quickly than hot air, and the steam steriliser could be used for other purposes, and is very convenient. However, it is a clumsy and slow method, requiring between thirty and forty minutes, and steel instruments rust very easily in steam. Boiling water or other fluids—and of the last glycerine and oil—have been proposed, but boiling glycerine gives off a horrible smell, and oil or any other fat is not desirable, because it is said to form a protective covering. The only satisfactory boiling fluid is water, which is an excellent disinfectant and can be procured at very short notice; and instruments which are boiled for five minutes are, as a rule, perfectly sterilised. Like steam and hot air, boiling water rusts steel instruments or covers them with black spots, but this drawback is easily obviated by adding a very small quantity, only 1 per cent. of some alkaline substance to the water, and for this purpose chalk, common salt, caustic soda, &c., have been proposed; but Bergmann showed that common household soda was, after all, the most efficient and satisfactory, and 1 per cent. completely prevented rusting. Moreover, it was further shown that the sterilising power of the boiling water was much increased by the addition of the soda. Thus this method claims the advantages of quickness, certainty, simplicity, convenience and the ease with which the necessary appliances can be procured.

### Sick Diet: Milk.

By a Trained Nurse.

### MILK MENUS.

It is important to bear in mind that barley water readily decomposes, that it needs to be fresh at least once daily, and that if it be heated to boiling point after it is once made, it is subject to rapid fermentation. Doubtless this combination of trouble in the making of barley water is the reason why lime water is so much more used. This is so easy to pour from a bottle. But the good nurse will not consider that the word "trouble" has any place in her vocabulary, and she will cheerfully prepare the barley water to add to her patient's milk, remembering that milk and barley water is infinitely nicer and more wholesome than is milk and lime water. In hot weather barley water should always be kept on ice. The proportion of barley water must vary with the digestive power of the patient. In ordinary cases one ounce of barley water is needed for each tumbler of milk. In extreme cases of difficult milk digestion half barley water and half milk must be used. Mixing the cold milk with hot barley water is a good plan, and gives a nice flavour to the milk. Where the digestion is good milk may be added in place of water to the barley, after the first boiling. But digestive difficulty will not always be so simply solved as by the addition of barley water to milk. I often find that this formula is necessary in order to make the digestion of milk a comfortable process: 2 ozs. of boiled cow's milk, 2 ozs. of barley water, 2 ozs. of hot distilled water. It will be seen that this is not a very nourishing food, but a small quantity of nourishment digested is worth oceans of nourishment which is given but not assimilated. In cases that will bear it a teaspoon of cream may be added to above.

But even the high dilution of the milk in this formula is not always sufficient to ensure its digestion. It will then be found an excellent plan to mix one part boiled cow's milk, one part barley water, and one part whey, with or without a little fresh cream, according to the condition of the digestion. This last formula is most digestible and satisfactory.

It will be noticed in the first recipe that hot distilled water is recommended for diluting the milk. Long experience has shown me the enormous advantage obtained by the use of distilled water for patients with typhoid or any gastric, hepatic, or intestinal troubles. In these cases I invariably obtain supplies of distilled water, and allow no other kind for the patient to drink. Even the most dyspeptic are able to enjoy their cup of tea when this is made with distilled water, and infused for two minutes only. In some extreme cases of gastric irritability I have been ordered by the doctor to make the barley water with distilled water. This sounds somewhat extravagant, but if distilled water be obtained in a large quantity at a time, it is by no means expensive. And after all, it is more extravagant to let the sick person suffer and thus prolong his gastric difficulties. There is no question that cases of typhoid, peritonitis, gastric ulcers, and all kinds of stomachic diseases suffer much from the ill-effects of chalky and "hard water." The extreme chalkiness of the water in some districts renders it very unsuitable for use by those suffering from gastritis or any other form of mucous membrane inflammation or irritability.

I am taking it for granted that, as a matter of course, the nurse will use boiled milk in all the formulæ. Raw milk is not only a heavy, indigestible fluid, but it presents many dangers from its power of absorption. This may be readily tested—if necessary—by placing an uncorked bottle of turpentine in close proximity to a tumbler of milk. Very soon the flavour of the turpentine will have communicated itself to the milk.

Even the flavour of a cigarette smoked in a room may be detected in any uncovered milk at hand. Boiling milk not only minimises germ danger, but it causes the formation of an "easier" curd for the digestion to deal with. Many persons object to the unpleasant taste of boiled milk. This is lessened by the skimming of the milk before boiling, and the addition after the process of the fresh cream. A pinch of salt is liked by many, while the nurse may provide an infinite variety of flavourings to prevent monotony. Half a teaspoon of meat extract, with pepper and salt, makes a pleasant vehicle for milk, and is liked thus by many persons who would not take the milk in its natural form. The nurse will invariably have to combat the strong superstition existing in most people's minds that "boiled milk" is more constipating than raw milk. The truth is that raw milk is often relaxing, because it is apt to produce digestive disturbances. Many babies for this reason, and some adults, suffer constantly from diarrhœa when taking unboiled milk. Nurses must remember that constipation is a common accompaniment of a fluid diet, for the simple reason that when living on liquids there is a very small amount of solid residue in the intestines. Some bulk in food has the effect of causing bowel activity, and this favourable factor is missing when the patient lives entirely on fluids. A few teaspoonfuls of strong bran water added to each glass of milk will help to overcome its constipating qualities; and in cases where this is permissible, the strained juice of stewed French plums (the quantity must necessarily vary with the age and condition of the patient), given the last thing at night in an aërated water, will frequently produce a satisfactory motion in the morning.

### Milk and Lime Water.

Lime water is added to milk for the same reason as barley, gelatine, or isinglass are used, because the addition of any of these mechanically separates the casein so that it coagulates into smaller flocculi than otherwise.

It frequently happens that, in order to make a milk diet digestible, the dilution of the milk with aërated waters and lime water is such that the quantity of fluid given is sometimes enormous. In typhoids this is very noticeable, and ten ounces at a time are often given every three hours. Now typhoid patients as a rule do not easily bear so much fluid given at one time, and it frequently appears to have the effect of seriously "hampering" the digestion. A good plan to obviate this is to add twenty minims of saccharated lime to one ounce of distilled water or whey, and add this to four ounces of pure, boiled milk. In this way the nourishment is much smaller in quantity and is generally well digested. In pleurisy, where it is not desirable that large quantities of fluid be taken, this plan proves very useful.

Ordinary lime water by no means improves the flavour of milk, but aërated lime water is very pleasant indeed. Many patients are extremely fond of an admixture of sherry and milk, but often have to forego it on account of the acidity it produces. In such a case it is well to try how equal parts of sherry, milk, and lime water act. In most cases the addition of lime water entirely counteracts the tendency of sherry to produce acidity.

### Condensed Milk.

Many patients are able to digest condensed milk much more easily than fresh cow's milk. The coagula formed by condensed milk are coarser than those produced by fresh milk and barley water, but in practice the condensed milk proves far more digestible, and is often most useful in tiding over gastric trouble. The unsweetened kind should always be used in the sick-room, and malt extract may be used with advantage as a sweetener. In all suitable cases a teaspoon of fresh cream should be added to each half-pint of the condensed milk solution, for condensed milk is usually deficient in fatty material.

### Post-Graduate Clinics for Nurses.

By a Trained Nurse.

### PRACTICAL POINTS IN LUNG DISEASES.—I.

The duties and responsibilities of nurses are ever widening out, and medical men more and more rely upon their nursing staffs to give trustworthy and intelligent help in every branch and new development of scientific treatment.

In these days of bacteriology and the microscopic examination of the sputum for the detection of the tubercle bacillus, and for diagnostic purposes generally, it is of the utmost importance that the nurse should be equipped with practical helpful points as to the collection and the keeping of such sputum. With regard to this, it is an important point to bear in mind that the mucus coughed up in the early morning, before substantial food has been taken, will not be only more clear of extraneous materials, but will be more typical of the condition of the lung than that taken at any other time.

Particles of food in the sputum are apt to prove confusing to the bacteriologist, and I have heard one or two eminent practitioners wax eloquent over the shortcomings of a nurse who had sent them a specimen of sputum containing a grape-stone or two, and other such foreign bodies, which entailed additional work.

The nurse after reassuring herself that only the sputum is present in the spittoon, should empty this into a tall glass—a clear white flower specimen glass serves excellently—and this should be kept carefully covered till it reaches the doctor's hands. Nurses cannot too often be reminded of the possible dangers present in sputum, especially when this is fœtid or tuberculous. Loose little gauze bags filled with medicated wool—pine wool serves admirably—form an excellent medium for receiving the sputum of phthisical patients, and such bags should be burnt immediately after use.

Many phthisical patients, however, insist on using pocket handkerchiefs, lest the use of wool bags such as described should call attention to their condition. In these cases the nurse must take care that the sputum does not dry on the handkerchief, for it is the dry sputum which holds such dangerous possibilities. Handkerchiefs used in purulent and phthisical cases should be put into a jar containing a solution of sanitas, thymol, or any other of the disinfectants which are not injurious to linen.

The handkerchiefs should be entirely covered by the liquid, and before being sent to the wash must be further disinfected by scalding with boiling water. Apart from the possible danger to others, the use of handkerchiefs prevents that watchful examination of the sputum, which many doctors impress on their nurses as being so necessary. Since the dry sputum is fraught with danger to the healthy, it is important that spittoons should invariably contain a liquid disinfectant. When expectoration is profuse and fœtid, iodine is one of the most pleasant and effectual disinfectants which can be employed.

An admirable form of hygienic spittoon can be obtained, which consists of a water-proof paper case fitting into a metal frame. The paper case is capable of holding a liquid disinfectant, and the whole may be burnt and a fresh case put in when necessary. The cost of the interior paper case is small, and it affords an economical protection against the infectiveness of the tuberculous germ.

Nurses frequently manufacture stiff paper or cardboard spittoons, the drawback to these obviously lying in the fact that they are not capable of holding a fluid disinfectant. If ordinary disinfectant spittoons are used, the utmost cleanliness is necessary. Some nurses think, in dealing with the china utensils of the sick room that disinfectants may be substituted for the housewifely soap and soda. In a point like this the domestic instincts are most valuable, for a nurse possessing these will take care in cleansing spittoons and all invalid vessels that boiling water and soda forms an invariable accompaniment to all disinfectants. In phthisical cases, and in cases where expectoration is fœtid, spittoons will need constant emptying and ceaseless cleansing. In a gangrenous condition of the lung, and cases attended with purulent mucus, as in some forms of phthisis and empyema, the room of the patient will require constant disinfection, by the use of vaporisers, blotting paper soaked in tincture of iodine and large towels dipped in disinfectants and hung about the room. Pill boxes full of iodine chips and with perforated lids are useful near the beds of such cases. For the purpose of room disinfection in lung cases disinfectants possessing little odour should be used. Sanitas, Condy, and bichloride of mercury answer better in these cases than do strongly smelling substances, for the latter frequently destroy the patient's appetite, and produce symptoms of nausea. Eucalyptus and terebene preparations, however, often prove most pleasant and efficacious in the disinfection of rooms occupied by lung patients. Iodine does not seem to have the effect of producing nausea or destroying appetite. It may, however, cause irritation of the mucous membranes, and, if so, must be discontinued. Disinfection of the room is not only advantageous to the patient by preventing him from re-absorbing poisonous products, but it is an essential and necessary protection to his nurse and to his friends.

Cases on record show that phthisis has been directly inoculated by the accidental breakage of a spittoon, and the consequent entry of the germ by direct contact with the blood of the individual unfortunate enough to break the spittoon; so that nurses in charge of phthisis and bad lung cases must exercise great caution.

The nurse must notice the characteristics of the sputa, whether ropy, stringy, or showing "casts." It is also important in all lung cases to note and report to the doctor any tinging of the mucus with blood. In bronchitis mucus stained or streaked with blood appearing frequently may denote incipient tubercle, so that such a symptom reported early to the doctor may prove of the utmost diagnostic value. After blood-stained mucus is coughed up the patient should be kept recumbent until the doctor has an opportunity of seeing him and giving further instructions. With regard to the bringing up of blood after or with mucus, it is important to notice whether this comes up naturally and quietly, or after a prolonged and exhausting fit of coughing. A point needing observation, too, is whether the blood has any odour beyond that normally possessed by blood.

### Death in Our Ranks.

It is with much regret that we record the death of Miss Ellen Thompson, one of the nursing staff of the Strangers' Hospital, Rio de Janeiro, on March 4th, from yellow fever. Miss Thompson was trained at the Nightingale School, St. Thomas's Hospital, in 1882; was staff nurse there from 1883 to 1887. She was matron at the Brighton College from 1887-93; head nurse at the British Hospital, Buenos Ayres, from 1893-96; and of the Strangers' Hospital from 1896 until death. Miss Thompson became ill on February 28th, from influenza, as was then supposed; two days later symptoms of virulent yellow fever showed themselves, and on the fifth day she died, and was buried in the evening. The unfortunate lady was an accomplished nurse, and much beloved by all who knew her. Her loss is greatly felt by her fellow nurses.

### The Hotel for Nurses.

14, ST. ANDREW'S CHAMBERS, WELLS STREET, OXFORD STREET, W.

EVERYTHING in the Hotel for Nurses in Wells Street is clean and pretty, the rooms though small are well furnished, and are decorated with light fresh papers and paint, whilst the arrangements for the convenience and comfort of those frequenting it seem well thought out. The position is central, being within a few minutes' walk of Regent's Circus. The accommodation offered varies from rooms containing two beds to a bed-sitting-room with the use of dining and drawing rooms large enough for about eight or ten persons. Judging by the tea table ready spread at the time of our impromptu visit, the food is plentiful, good, and the price cheap. The table was spread with a clean cloth and adorned with plants in ornamental pots and white china baskets of moss and flowers. In one corner of the room there was a book case furnished with a good supply of current literature. The furniture was of stained green wood—simple, adequate, and in good taste. That in the drawing-room was in the same style, and on the walls of both were hung engravings of the works of good artists. The bed-sitting-rooms have art carpets on the floors, and are fitted with brass bedsteads, easy chairs, writing tables, wardrobes, and marble-topped dressing chests. The bath-room is in accordance with modern sanitation. It is lined half-way up the walls with brown glazed bricks; the floor is terrazzo, the bath is of enamelled iron and uncased, and there is also a fitted lavatory. The staircase is distinctly narrow. Miss E. Debenham, of 1, Fitzjohn's Avenue, N.W., the lady superintendent, will supply a copy of the rules upon application. The chambers may also be inspected in the mornings between half-past eleven and half-past twelve o'clock. A double-bedded room by the year costs its tenants £20 apiece, a single-bedded room costs £25, and a bed-sitting-room £30. By the day the price is respectively 2s. 6d., 3s., and 3s. 6d., and by the week 12s. 6d., 15s., and 17s. 6d. The board is reasonable: 4d. for tea, 6d. for breakfast, 8d. for supper, and 1s. 2d. for a dinner of three courses.

### Everybody's Opinion.

[Correspondence on all subjects is invited, but we cannot in any way be responsible for the opinions expressed by our correspondents. No communication can be entertained if the name and address of the correspondent is not given, as a guarantee of good faith but not necessarily for publication, or unless one side of the paper only is written on.]

### *RE* LINSEED MEAL POULTICES.

MRS. STEELE writes: I have great pleasure in replying to your correspondent's query as to the best way to make a good linseed poultice, having found the following mode to be of great comfort, especially with infant cases of severe illness, where any weight was objectionable: Procure half a yard of unbleached cotton wool at the drapers, which will be found to be enclosed between a thin substance resembling skin. Pull apart in the middle, when you will have a yard of soft wool with outside skin; cut this into squares twice the size required for a poultice. Make a soap dish or basin hot and put in sufficient linseed meal for once only, mix with boiling water to a smooth substance neither hard or sloppy, drop on to the cotton wool square, leaving the edges clean, and you will have a perfect poultice with warmth and cleanliness, the outside edges of wool comfortable to the patient, and the skin of the wool retaining warmth and preventing moisture escaping.

### Presentations.

MISS GREENHOUGH SMITH, the retiring matron of the Bristol General Hospital, was presented on Friday, March 25th, upon her departure, with an easy chair from the residents past and present, with an oak writing table and chair from the nursing staff, and with a handsome inkstand from the servants and porters. Miss Smith is very popular, and she carries with her the good wishes and affection of all who have been under her guidance.

### The Book World for Women and Nurses.

[We invite Correspondence, Criticism, Enquiries, and Notes on Books likely to interest Women and Nurses. Address, Editor, THE HOSPITAL (Nurses' Book World), 28 & 29, Southampton Street, Strand, London, W.C.]

PERPETUA. By S. BARING-GOULD. (Isbister and Co. 1897.)

To describe truly, in the nineteenth century, the daily life of the third century is impossible. It is, therefore, not wonderful that this volume, in which Mr. Baring-Gould's theme is the legend of Saint Perpetua, martyred at Nimes in 213, should be rather dull reading. Nor is the story itself anything but commonplace. Every seven years a victim must be thrown into the fountain of Nimes to propitiate the god Nemausus. In A.D. 213 the lot falls upon a Christain maid, Perpetua. She is thrown into the holy basin, but a Roman youth, Æmilius Varro, plunges in, saves her, and, for a time, succeeds in hiding from the pursuit of the priests. In the end, however, she is recaptured and, refusing to save her life by sacrificing to the Pagan god, is broken on the wheel in the arena. Just as she is about to die a miraculous fall of snow descends and hides her from the eyes of the spectators. For an hour Æmilius kneels besides her. "Then suddenly the clouds parted, and the sun poured down a flood of glory over the dazzling white oval field, in the midst of which lay a heap of whiteness, and on a face as of alabaster, inanimate, and on a kneeling, weeping man, still with reverent finger sweeping away the last snowflakes from eyelash, cheek, and hair, and who felt as if he could thus look and kneel and weep for ever." It is superfluous to say that this story, in the experienced hands of the author, is told in vivid and readable language. But, after all, we cannot help thinking that it will add little to his reputation. It will, however, be useful as a Sunday-school prize-book; and we suspect that the learned author has himself had this in view. Many of the pages remind us of Smith's Classical Dictionary, or of some handbook on the elements of Church history. The guests sup in the *triclinium*. They wash their hands in the *impluvium*. The magistrates of Nimes "sent *vigiles* (*i.e.*, the watch)." *Carcer* means a prison. "A Roman prison consists of several parts"; and so on. This will be interesting and instructive to some intelligent schoolboy; and the passages which depict the phases of thought which pervaded the world during the first centuries of the Christian era are rather trite. "The period when Christianity began to radiate through the Roman world was one when the traditional Paganism, with its associated rites, that had contented a simpler age, had lost its hold on the thoughtful and cultured. . . . Men craved to know. . . . Christianity, meeting a wide-felt want, spread rapidly, not only among the poor and oppressed, but extensively among the cultured and the noble." Nevertheless, stale as all this may be, the author has probably succeeded as well as any of his numerous predecessors in the same field in the attempt to draw a picture of a state of society so remote from our own day that it is impossible for us to realise it. We prefer to wander with Mr. Baring-Gould through more familiar scenes.

### Minor Appointment.

ISLE OF THANET UNION WORKHOUSE, RAMSGATE.—Miss Edith Mary Mellody was appointed Superintendent Nurse of this establishment on March 31st. She was trained at the Parish Infirmary, Brownlow Hill, Liverpool, and has been charge nurse both at the Eastern Fever Hospital and at Toxteth Infirmary, Liverpool, where she was also night superintendent.

LANCASTER UNION INFIRMARY.—Miss Margaret Walker was on March 29th appointed to the post of Superintendent Nurse of this infirmary. She was trained at Huddersfield Infirmary, and afterwards held appointments at Carlisle, and at Fulwood, near Preston.

CITY OF WORCESTER ISOLATION HOSPITAL.—Miss E. V. Godfrey, who was trained at Monsal Fever Hospital, and who was subsequently charge-nurse of the Leyton Isolation Hospital, has been appointed Charge-Nurse of the scarlet fever block of the above hospital.

### Nursing in Paris Hospitals.

### A.—LAY NURSES.

### VIII.—The New Trade Unionism.

The most recent and not least interesting development of the service of lay nursing in Paris is the awakening of the nurses themselves to an aspiration of self-help in bettering their own condition, and, it is to be hoped, their capabilities at the same time. That the nurses have had many grievances to complain of is something beyond dispute. I have hitherto shown how, in the question of pay, allowances, and lodgment, many things exist to breed dissatisfaction in the rank and file.

This state of things has been taken advantage of by various trade union propagandists to stir up the nurses to make numerous demands upon their employers, the holders of the public purse-strings, and what is practically a nurses' trades union has been formed. It has not yet adopted the name of trades union, but contents itself with the title of Groupement du Personnel Secondaire de l'Assistance Publique à Paris. By far the most active in this new crusade is Madame Duvignaud, who holds the two offices of vice-president and recording secretary. The ordinary weakness of humanity is probably responsible for the fact that the new trade union has hitherto not entirely busied itself with the grievances of minor classes, who are subject to so severe special hardships, or to details of inadequate or insufficient accommodation or allowances, but has engaged in a general demand for increased wages all round.

I have stated that the present scale of pay was only ordered as lately as December, 1896. This was voted by the Municipal Council upon a direct demand, elaborately formulated by the new trade union organisation. This rather remarkable document, addressed from a large body of public servants to the representatives of the people, included a long list of grievances and remedies demanded. These demands are under seven heads. The first head was subdivided into three sections, of which the first section asked for equalisation of wages, the second for the wages to be increased to the same scale as the other employés of the Assistance Publique, and the third section asked for the pensions to be made equal to those of the asylum employés under the Department of the Seine. The second head was a request for the equalisation of food allowances. The third was for equalisation of fuel and light allowances. The fourth head was that, where husband and wife are both nurses and only occupy one lodgment, they shall be allowed money indemnity for another. The fifth head was a demand for the establishment of the central employment bureau which has since been instituted. The sixth head was, perhaps, the most startling of all, being a demand for the enforcement of a rule that two-thirds of all promotions must be for seniority, and only one-third for special merit, or as they neatly put it "au choix." The seventh and last head of this elaborate demand of the nurses was that the same regulations shall be in force in all hospitaliary establishments under the control of the Assistance Publique.

That the new trade union is by no means modest in making itself heard is shown by its authoritative intervention in the question of male and female nurses. On this subject the demand of the association is formulated as follows: "One of the most ardent wishes of the secondary staff of all grades is to see the male nurses for the men, and female nurses for the women. It is immoral to see women forced to make certain dressings, and to hear all the day long improper remarks by their male patients." This is only one of a long series of demands for what is styled the "Campaign, 1897-1898." As a result of the enforcement of this rule would be the replacement of many women by men, it was, perhaps, an attempt on the part of men to obtain more situations rather than chivalrous regard for the women which has inspired the request. One other of the demands is a renewed request for the allowance of double lodgment pay to husbands and wives both in the service, so that they may be able to pay for accommodation outside. Another curious claim is for quite grandiose funeral ceremonies for all nurses dying in office.

The meetings of the union have been mostly held at the various hospitals with considerable flourish of trumpets, they being usually attended by the hospital director and some members of the Municipal Council, and much eloquence has usually been expended.

Although the hospital directors have attended the meetings of the "Groupement," and made complimentary speeches, I gather that they generally look with considerable distrust as to the ultimate outcome of this new movement. There is a lurking suspicion that the whole thing may possibly be fostered by municipal politicians for cheap electoral capital. Perhaps for this reason the male element, with its votes, is so much to the fore, although women are the preponderating element in the nursing system. One hospital chief said flatly that those that were patronising the new organisation to-day were sowing the wind and would reap the whirlwind. Another cautiously remarked that if the present association ended in an avowed trade union, a strike would be the result. Of course, nothing more scandalous can be imagined than a strike of nurses.

The "Groupement" has a weekly organ, *L'Infirmier*, edited by the secretary, M. Lheritier, with the honorary assistance of M. Paul Strauss, the Senator, and M. Chauvière, Deputy. It is only fair to say that both paper and organisation attest the zeal and devotion of the secretary, and are patronised not only by MM. Strauss and Chauvière, but by some of the leading medical hospital staff, such as Professor Raymond and Dr. Charcot, the younger.

I will conclude this account of the new union by giving its objects as set forth in the prelude to its constitution: "The object of the Groupement of the secondary staff is to affirm in our body the principles of solidarity, being formed to aid the family in the painful trials of illness and death; to assure to the sick the consolations and moral support which is nearly always wanting among those of us who are in adversity; to study and to try to successfully solve all questions concerning the amelioration of our condition, the improvement of our administrative situation, and to aid each member by all the means in our power, and in all the circumstances where intervention may be judged necessary." All this sounds quite innocuous, but *qui vivra verra.*

Edmund R. Spearman.

### For Reading to the Sick

EASTER.

**Verses:**

'TWAS at the matin hour, early, before the dawn,
The prison doors flew open, the bolts of death were drawn.
'Twas at the matin hour, when prayers of saints are strong,
Where, two short days ago, He bore the spitting, wounds, and wrong.

From realms unseen, an unseen way the Almighty Saviour came,
And following in His steps an angel armed in flame.
The stone is rolled away, the keepers fainting fall;
Satan's and Pilate's watchmen, the Day has scared them all.

The angel came full early, but Christ had gone before,
The Breath of Life, the living Soul, had breathed itself once more
Into the sacred Body that slumbered in the tomb,
As still and lowly as erewhile in the undefiled womb.

—*John Keble.*

EASTER EVE.

Lo, as we come with buds of spring
To deck Thy House of Prayer
We muse on those who once did bring,
Their spices rich and rare,
To dew Thee with their offering,
And lo, Thou wast not there.

Nay, Lord, not in the silent tomb,
There buried lies our sin,
There like a seed which dies to bloom
Does our new life begin;
Thou from the gates of death art come,
Eternal Life to win.

Not yet, not yet, we do not know
The joy to be revealed;
Our hearts bereft are numbed with woe,
The sepulchre is sealed,
And Thou art lying bruised and low,
Who hast the whole world healed.

Still pressing like a weary load,
Our sins lie heavily,
The sins that nailed the Son of God
To the accursed tree;
Yet must we sadly take the road
That leads from Calvary.

Only to mourn a little space,
With tears of penance sore,
Then seek Thee in Thy Resting-place,
Thy Body to weep o'er;
And hear Thy Voice, and see Thy Face,
And never lose Thee more. —*E. M. L.*

**Reading.**

Alleluiah! Alleluiah! Alleluiah!

Very early in the morning . . . when it was yet dark, the angel of the Lord descended from heaven, and came and rolled back the stone.—*St. Matthew* xxviii. 2.

An angel in a sepulchre is a very strange sight; what doth an angel there? Indeed, no angel ever came there till this morning, Not till Christ had been there; but now He has left there *odorem vitæ*, and changed the grave into a place of rest. Why not the bodies in the grave to be in Heaven one day, as well as the angels of Heaven to be in the grave this day?—*Bishop Andrewes.*

Our Lord, in His unbounded love, has taken great pains to teach us that death is to be considered but a sleep, that it is to be in our minds but associated with sleep, and to be lightened and cheered by that association . . . showing us thereby His desire that we should look on death as but a sleep, in ourselves and others.—*Isaac Williams.*

### Notes and Queries.

**The contents of the Editor's Letter-box have now reached such unwieldy proportions that it has become necessary to establish a hard and fast rule regarding Answers to Correspondents. In future, all questions requiring replies will continue to be answered in this column without any fee. If an answer is required by letter, a fee of half-a-crown must be enclosed with the note containing the enquiry. We are always pleased to help our numerous correspondents to the fullest extent, and we can trust them to sympathise in the overwhelming amount of writing which makes the new rules a necessity. Every communication must be accompanied by the writer's name and address, otherwise it will receive no attention.**

*The Hospital Convalescent Fund.*

(8) Is a nurse 50 years of age and a chronic invalid eligible for help from the Hospital Convalescent Fund, and if so, to whom should she apply?—*E.*

As the aim of the Convalescent Fund is to enable sick nurses to resume work, a chronic invalid is not eligible. Apply to some of the numerous homes (see "Burdett's Hospitals and Charities" for list).

*Paying Measles Patient.*

(9) Is there in the metropolis an institution which will receive patient attacked with measles for nursing and medical attendance at an inclusive fee of 40s. to 50s. per week?—*Stretcher.*

There are occasionally vacant beds for measles at the London Fever Hospital, Liverpool Road, Islington, at £3 3s. a week. There are no others in the metropolis.

*Emigrant Probationers.*

(10) Can two sisters, age 20 and 23, enter a hospital in Australia as probationers? Please tell us in what way we can do so.—*Two Orphans.*

There are several excellent training schools in Australia. "Two Orphans" should consult Miss Lefroy, The United British Women's Emigration Association, Imperial Institute, S.W., as to the part of Australia that would be most suitable to their health, and then make arrangements to go out under the auspices of the society. If they will let us know their destination, we can give them the address of a training school where they can apply.

*A Mill Hand.*

(11) Will "Minister" send name and address? It is against our rules to reply to queries without them. They are not for publication, but are proofs of good faith, without which we cannot act.

*How to Become a Nurse.*

(12) I am desirous of becoming a nurse, and would like to know where to train and what there is to pay.—*Anxious.*

See Honnor Morten's book, "How to Become a Nurse" (The Scientific Press, 2s. 6d) It is in many cases unnecessary to pay anything.

*Near Sight.*

(13) I am 25 years of age and very fond of nursing; would being a little short-sighted disqualify my entering a training school as probationer? I would wear glasses if necessary.—*R. E. H.*

The medical man who will examine your eyes if you become an accepted candidate can best answer this query. In any case you ought to wear spectacles if necessary.

*Homes of Rest.*

(14) Will you kindly give me a list of homes of rest for ladies where payment is not required?—*Nurse Beatrice.*

Buckmaster Memorial Home, Broadstairs, three weeks free; apply G. F. Billinge, 82, Angel Road, Brixton, S.W. House of Rest for Ladies, Wokingham, Berks; apply Miss Blair, Heatherly, Wellington College Station, Berks. Wightwick Manor Farm, Wolverhampton; apply Mrs. Theodore Mander.

*Who Owns Uniform?*

(15) I have been a district nurse for 15 months; am I entitled to keep uniform, which consis s of two bonnets and a cloak?—*B. R.*

The uniform is the wearer's property on completion of twelve months' service (in some institutions at the end of six months). In small private hospitals the nurses have sometimes to give it all up on leaving. In some asylums and hospitals of the M.A.B., instead of three dresses and six aprons, &c., being issued yearly, these articles are condemned as they wear out and replaced by new ones—the most economical way.

*Uniform for Sale.*

(16) I have a navy blue winter cloak in good condition and a new bonnet which I should like to part with. Can you help me?—*L. G.*

We have not a department for sale and exchange of costumes.

*Diagrams.*

(17) Where can I obtain a large varnished canvas wall diagram of the circulatory system?—*Lady Superintendent.*

Large physiological diagrams may be obtained from the St. John Ambulance Association, The Chancery, St. John's Gate, Clerkenwell, London, for 15s., amongst which is one on the heart and circulation of the blood.

*Hair Dye.*

(18) Can you tell me how to make a cheap hair dye for a poor man who is out of work eight months? Aged fifty-four.

### Wants and Workers.

A. S. W., 283, Amhurst Road, Stoke Newington, wishes to buy a second hand copy of a book of medical terms.

